# Association between landscape transformation and the Chagas disease vector dynamics in a rural area with continuous surveillance and control

**DOI:** 10.1186/s13071-025-06849-1

**Published:** 2025-06-02

**Authors:** Leonardo Sandon, Diego Weinberg, Manuel O. Espinosa, Marcelo C. Abril, Roberto Chuit, Ximena Porcasi, Maria V. Periago

**Affiliations:** 1 Fundación Mundo Sano, Buenos Aires, Argentina; 2 Comisión Nacional de Asuntos Espaciales (CONAE), Córdoba, Argentina; 3 Consejo Nacional de Investigaciones Científicas y Técnicas (CONICET), Buenos Aires, Argentina

**Keywords:** Chagas disease, *Triatoma infestans*, Deforestation, Land use change, Surveillance, Vector control, Spatial analysis, Gran Chaco, Argentina, Zoonotic disease

## Abstract

**Background:**

The Gran Chaco Region of Argentina, a hotspot for neglected tropical diseases (NTDs) including Chagas disease (CD), has undergone significant landscape transformations due to deforestation and agricultural expansion. These changes have altered the dynamics of *Triatoma infestans*, the primary vector of *Trypanosoma cruzi*, the causative agent of CD. This study investigates the association between environmental changes and vector infestation patterns in a rural area of Añatuya, Santiago del Estero, Argentina, under continuous surveillance and control since 2005.

**Methods:**

Entomological data from 502 households across 13 rural settlements were collected over a decade (2009–2019). Spatial patterns of triatomine infestation were analyzed using kernel density estimation (KDE), and relationships between infestation, land use changes, household density, and proximity to deforested areas were assessed. Random Forest models were employed to identify key predictors of infestation, incorporating variables such as deforestation percentage, distance to agricultural plots, and domestic animal density.

**Results:**

Infestation patterns were highly heterogeneous, with significant hotspots identified in settlements such as Lote 27, Lote 47, and La Salamanca. Household density and distance to main roads emerged as the most important predictors of infestation, with higher infestation rates observed in areas with lower deforestation and greater distance from agricultural plots. Deforestation reduced sylvatic reservoirs of *T. cruzi* but influenced domestic triatomine populations, particularly in areas with intermediate household density. Continuous surveillance and control efforts, including insecticide application and house improvements, led to a significant reduction in infestation rates over time.

**Conclusions:**

Landscape transformation plays a critical role in shaping *T. cruzi* transmission dynamics. While deforestation reduces sylvatic reservoirs, it also influences domestic triatomine populations, highlighting the complex interplay between environmental changes and vector ecology. Tailored control strategies that address both domestic and sylvatic cycles are essential for sustainable vector elimination. These findings underscore the importance of integrating environmental and spatial factors into CD control programs to achieve certification of transmission-free areas and reduce the burden of CD in endemic regions.

**Graphical Abstract:**

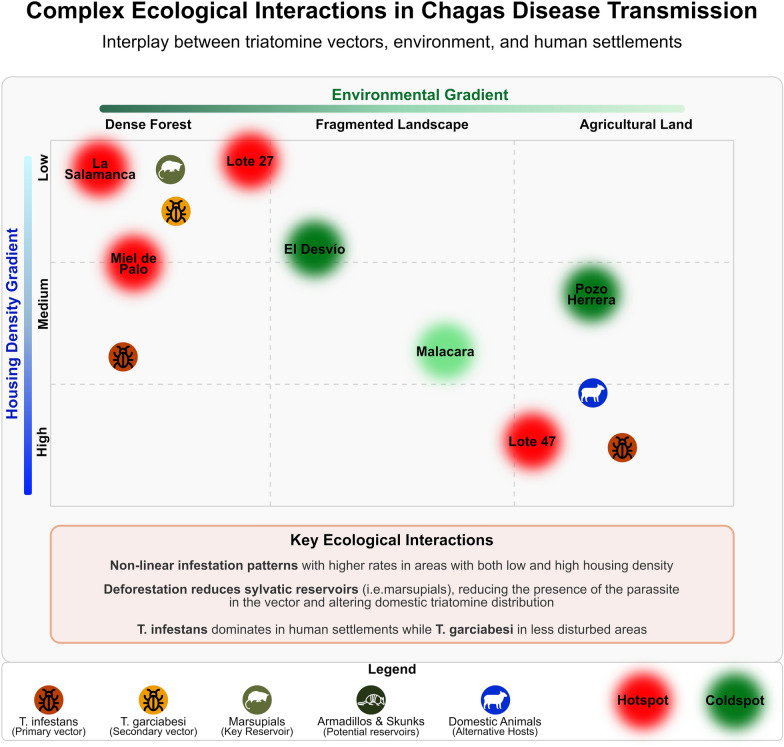

**Supplementary Information:**

The online version contains supplementary material available at 10.1186/s13071-025-06849-1.

## Background

Argentina is divided into 18 environmental regions, including the Dry Chaco and the Humid Chaco [1]. These two regions are part of the Gran Chaco Region, which houses a vast number of animal and plant species and extends to Bolivia, Brazil, and Paraguay. Approximately 60% of the native forests of Argentina are located within this region [2, 3]. Nonetheless, and despite having some areas that are currently protected, the region has been exploited since colonial times, leading to deforestation [2, 3]. Changes in land use due to agricultural expansion for cattle raising and crops occurred more recently, in the twentieth century, accelerating deforestation [3, 4].

There are 11 Argentinian provinces (out of a total of 24) located within the Gran Chaco Region, including Chaco; Santiago del Estero; Formosa; the north of Santa Fe, Cordoba, and San Luis; the east of Salta, Tucuman, La Rioja, and Catamarca; and the west of Corrientes [5]. In addition to being a region rich in flora and fauna, it is also considered a hotspot for neglected tropical diseases (NTDs), a group of infections caused by different agents that affect an estimated 2.7 billion people living in rural areas and in urban settings of low-income countries in sub-Saharan Africa, Asia, and Latin America [6, 7]. In this area of the Argentinian Gran Chaco, the presence of different NTDs has been reported, including Chagas disease (CD), caused by the parasite *Trypanosoma cruzi* and transmitted through different vector insects of the Reduviidae family, subfamily Triatominae [8].

CD is considered a zoonotic disease due to its sylvatic origin, and *T. cruzi* has been detected in approximately 180 different animal species, especially affecting a wide range of small mammals [9]. The main and most widespread vector of *T. cruzi* in South America is *Triatoma infestans*, thought to have originated in the inter-Andean valleys of Bolivia [10] or through multiple events of domestication in different areas [11]. Additionally, it has been passively dispersed with the movement of goods and humans [12], although not fully adapting to new sylvatic habitats. The distribution of this species, more closely associated with domestic environments, has been widely reduced due to different targeted control actions; several countries of the Southern Cone have already been certified for interruption of *T. cruzi* transmission by *T. infestans*, including Brazil, Chile, Paraguay, and Uruguay [13].

Currently, this species persists in the Andean valleys of Bolivia and the Gran Chaco Region [14]. In Argentina, *T. infestans* is highly adapted to the rural domestic environment, infesting households and peridomestic structures and feeding on domestic animals, including dogs, chickens, and farm animals [11]. Nonetheless, ten provinces from Argentina have been already certified as free of vector transmission by *T. infestans*: Corrientes, Entre Ríos, Jujuy, La Pampa, Misiones, Neuquén, Río Negro, San Luis, Santa Fe, and Tucumán [15]. These advances are probably a combined result of control activities and land use changes, given that the impact of human activities on the landscape can influence both the prevalence of infection in animal reservoirs, reducing their abundance and distribution, the presence, and prevalence of infection in vector species, and the likelihood of contact with humans [16–19].

Previous studies have explored the relationship between household characteristics (type of materials used and presence of peridomestic structures, among others), community involvement, and vector control to elucidate the factors involved in the presence of triatomines in domestic habitats [20, 21]. While large-scale studies have reported associations between the environment and the presence of triatomines, such as the normalized difference vegetation index (NDVI ) and temperature [22–26], changes in land use [11], or modification in the abundance of mammalian reservoirs for *T. cruzi* [16, 27–29], other studies suggest working at a smaller scale to understand factors that modulate focal transmission cycles [30–32]. For this reason, the study described herein focuses on the spatial pattern of infestation of households by triatomine bugs, assuming that the infestation process is homogenous over space, in a rural area of Santiago del Estero, not yet certified as free of vector transmission, Añatuya, and explore the relation between the modified environment and vector dispersion.

Santiago del Estero is a Chagas endemic province that is awaiting certification as free of vector transmission of *T. cruzi* and has advanced with certification in departments located in the south, bordering Santa Fe province, including Aguirre, Mitre, Rivadavia, Belgrano, Quebrachos, and Ojo de Agua [33]. Añatuya is within the Department of General Taboada, located to the north, and bordering those departments that have already been certified as free of vector transmission. Additionally, the rural settlements located around Añatuya have been under entomological surveillance and control for more than 10 years [34]. Therefore, an analysis of the factors associated with the spatial distribution of infestation through time should give us information on the effect of not only long-term vector control but also the effect of environmental changes and spatial or contouring factors such as house density, distance to main roads, changes in land use, and deforestation.

## Methods

### Study area

**Fig. 1:**
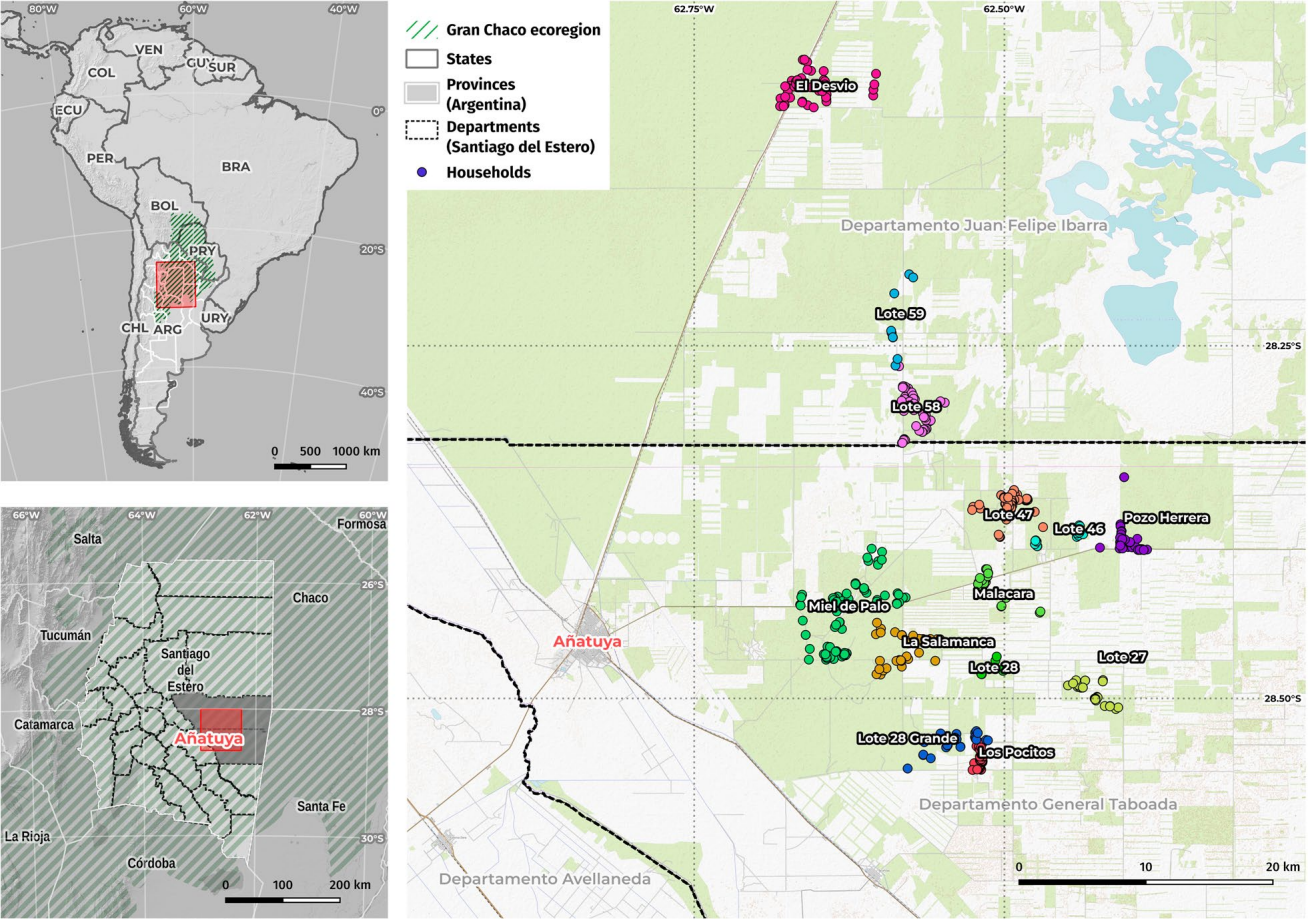
Maps showing the study area at different scales (continental, national, and local). The local scale includes the city of Añatuya, as well as the different rural settlements included in the study, located in the Departments of Juan Felipe Ibarra and General Taboada, Santiago del Estero Province, Argentina. The maps were generated using QGIS (version 3.39) with OpenStreetMap (OSM) data and the OpenTopoMap render style. Map data: © OpenStreetMap contributors, SRTM | Map design: © OpenTopoMap (CC-BY-SA)

In the current study, we first focus on the spatial pattern of infestation of households by triatomine bugs assuming a homogeneous space given the similarity in the household structure, socioeconomic background, and characteristics of the population in the study area. Then, we evaluate the relationship between changes in land use, using a 50 m buffer around each house, to explore the relation between the modified environment and vector dispersion in a rural area of Añatuya, Santiago del Estero, Argentina (Fig. 1). The city of Añatuya (28° 27′ 36″ S 62° 50′ 02″ W), located in the central department of General Taboada has been under entomological surveillance and control (S&C) for triatomines as part of a Chagas Disease program since 2002. Neighboring rural settlements from General Taboada and Juan F. Ibarra departments were added to the S&C program in 2005, including household sanitary improvement [34, 35].

In this study, entomological data from the last 10 years of program implementation (2009–2019) in 13 rural settlements composed of a total of 502 households, were used: El Desvío, Miel de Palo, La Salamanca, Lote 28, Pozo Herrera, Lote 46, Lote 47, Lote 27, Lote 58, Lote 59, Los Pocitos, Lote 28 Grande, and Malacara [34].

### Entomological and control data

Briefly, for the analysis in the current study context, household positivity was calculated as the number of times a household was positive either in the intra- or peridomicile, divided by the number of times the household was visited, as scheduled by the vector control program in place [34]. As part of the standard national control protocol, insecticide was applied to the intra- and peridomicile of triatomine-positive houses. Insecticide was applied by trained personnel not only in the infested house but also in surrounding houses, within a 500 m radius, regardless of the presence of triatomines, as per national guidelines [34–37].

### Environmental and land use data

The main source of environmental and land use data was obtained from a cartographic study based on Landsat imagery with a spatial resolution of 30 m. For the period prior to 2000, land cover changes were analyzed across three intervals (1976–1986, 1986–1996, and 1996–1999), while from 2000 onward, deforestation was monitored annually using cloud-free scenes primarily captured between November and January, corresponding to the dry season when most land clearing occurs [38]. The variables used include: (1) the percentage of transformation, calculated as the percentage of transformed surface in each buffer area, using a 2 km buffer around each household; (2) the average year of transformation using a raster with information on deforested plots, where the value of each cell represents the year in which deforestation occurred (weighed by surface area); (3) distance to agricultural plots, calculated as the average distance in each buffer area; (4) the perimeter of agricultural plots, calculated as the average perimeter of agricultural plots in each buffer; and (5) the density of domestic animals, calculated as the sum of animals recorded in each household (weighing the size of the different animals present in the area).

### Statistical analysis

To evaluate whether the spatial distribution of triatomine infestation in households was homogeneous in the study area, a spatial analysis based on the kernel density estimation (KDE) method was performed [39–41]. The KDE was applied using a bandwidth of 2 km and a quartic kernel function, which controls the degree of smoothing and the influence of each point over space. All the spatial variables were considered in the context of the distance of active dispersal (by flying or walking) of triatomine bugs and their immediate surroundings [42, 43]. Through this technique, a continuous spatial density map of positive households, calculated as the observed rate, was generated. At the same time, an expected density, calculated under the assumption that every household has the same infestation rate (assuming a homogeneous distribution), was used. This same concept of uniform distribution was used to measure the degree of insecticide application over the study area.

It is important to note that, although not all households were visited the same number of times, this method effectively standardizes the infestation rate, allowing even households with fewer visits to have comparable values to those visited more frequently. The difference between these densities, normalized by the standard deviation of the number of expected positive households, was used to generate diffusion maps that identify clusters of positive (hotspots) or negative (cold spots) households. A difference greater than zero between the expected and observed value constitutes a hotspot, while a value lower than zero constitutes a cold spot.

A standardized difference map between observed and expected, homogeneous over space, was generated using the following formula: *Sdif* = (*Obs* − *Exp*)/SD(*Exp*), where *Sdif* is the standardized difference, *Obs* is the observed value, *Exp* is the expected value, and SD(*Exp*) is the standard deviation of the expected values. Both observed and expected infestation maps calculated a 2 km buffer area around each household. Additionally, the KDE method was used to detect areas where the presence of domestic animals was higher or lower than expected under a homogeneous distribution. To analyze the significance of the KDE data, the GeoDa software [44] was used, calculating Moran’s *I* global and local indices (local indicator of spatial association [LISA]). Moran’s *I* global is a measure of spatial autocorrelation used to assess whether similar values are significantly clustered in space, while LISA allows for the evaluation of whether each cluster location is statistically significant. For each household, the statistical significance of the infestation value was calculated using the pseudo-*p* method, through a conditional permutation approach.

The smoothed data obtained from KDE over vector control actions and triatomine persistence were compared through the evaluation of the congruence of both variables across the study area. The relationship between the spatially smoothed KDE presence of triatomines, land use, and environmental variables was also analyzed.

### Random forest models

To better understand the correlations between spatial infestation patterns of triatomines, two random forest models (RFMs) were developed, with the dependent variable being the infestation spatial pattern (KDE). These models differ in the number and type of predictor variables included: Model 1 included only land use variables, while model 2 included both environmental and control-related variables. The statistical analysis was performed using RStudio software [45], and the random forest models were implemented with the Random Forest package [46]. To interpret the results of the RFMs, we focused on two key outputs: variable importance [47] and partial dependence plots (PDPs) [48].

Variable importance, quantified as “increase in node purity” (IncNodePurity), provides insight into the significance of each predictor variable in explaining the spatial patterns of triatomine infestation. IncNodePurity measures the total reduction in node impurity using Gini impurity attributed to a variable across all trees in the forest. Higher IncNodePurity values indicate that a particular variable plays a more critical role in determining infestation patterns. Additionally, PDPs were employed to visualize the marginal effects of individual variables on infestation risk. PDPs illustrate how the predicted probability of triatomine infestation changes as a specific predictor variable is varied, while all other variables are held constant. This allows for a detailed examination of whether the relationship between each environmental factor and the likelihood of infestation is linear and nonlinear or involves thresholds, providing a deeper understanding of the spatial dynamics at play.

## Results

### Triatomine infestation density

**Fig. 2:**
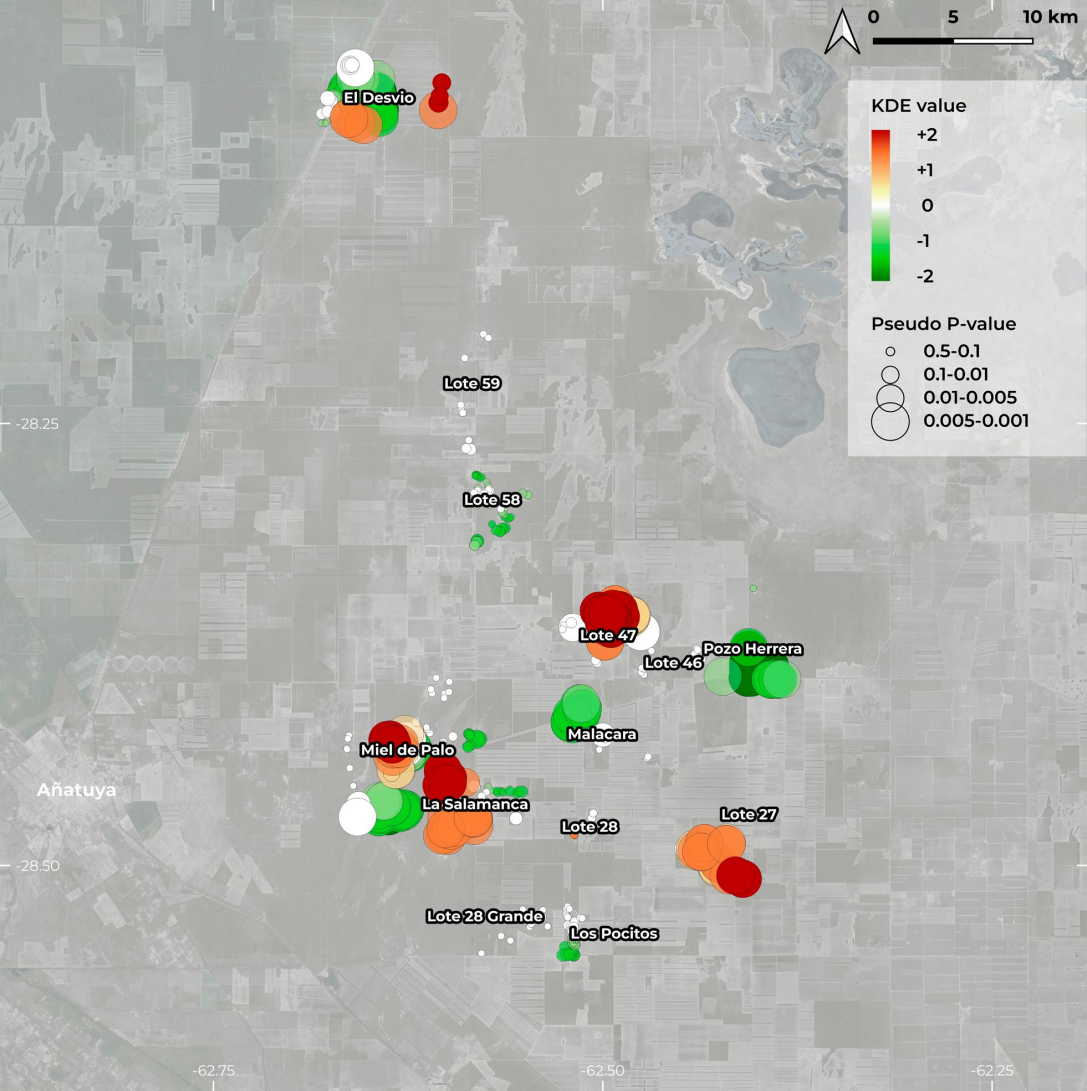
Map of the spatial distribution of triatomine infestation density (2009- 2019) in the rural settlements of Añatuya (Santiago del Estero, Argentina) included in the study. Red indicates values higher than expected, while green represents values lower than expected. The size of the circle indicates the significance value (pseudo *p*-value). Created with QGIS (version 3.39), with background satellite imagery from Google Maps © Google Maps, CNES/Airbus, TerraMetrics, 2024)

The KDE map obtained for the study area shows the distribution of infestation density in households across different rural localities (Fig. 2). Additionally, Moran’s *I* global index (0.733; *p* = 0.001) indicates the presence of a significant spatial pattern—in this case, households with similar infestation rates. This suggests that 80% of the households show values different from a homogeneous distribution, indicating that the distribution of triatomines is heterogeneous and nonrandom in the study area. Through the LISA index, statistically significant hotspots were detected in Lote 27, Lote 47, Miel de Palo, and La Salamanca (Fig. 2), where triatomine density was higher than expected. Meanwhile, in the localities of El Desvío, Pozo Herrera, and Malacara, significant cold spots were detected.

**Fig. 3:**
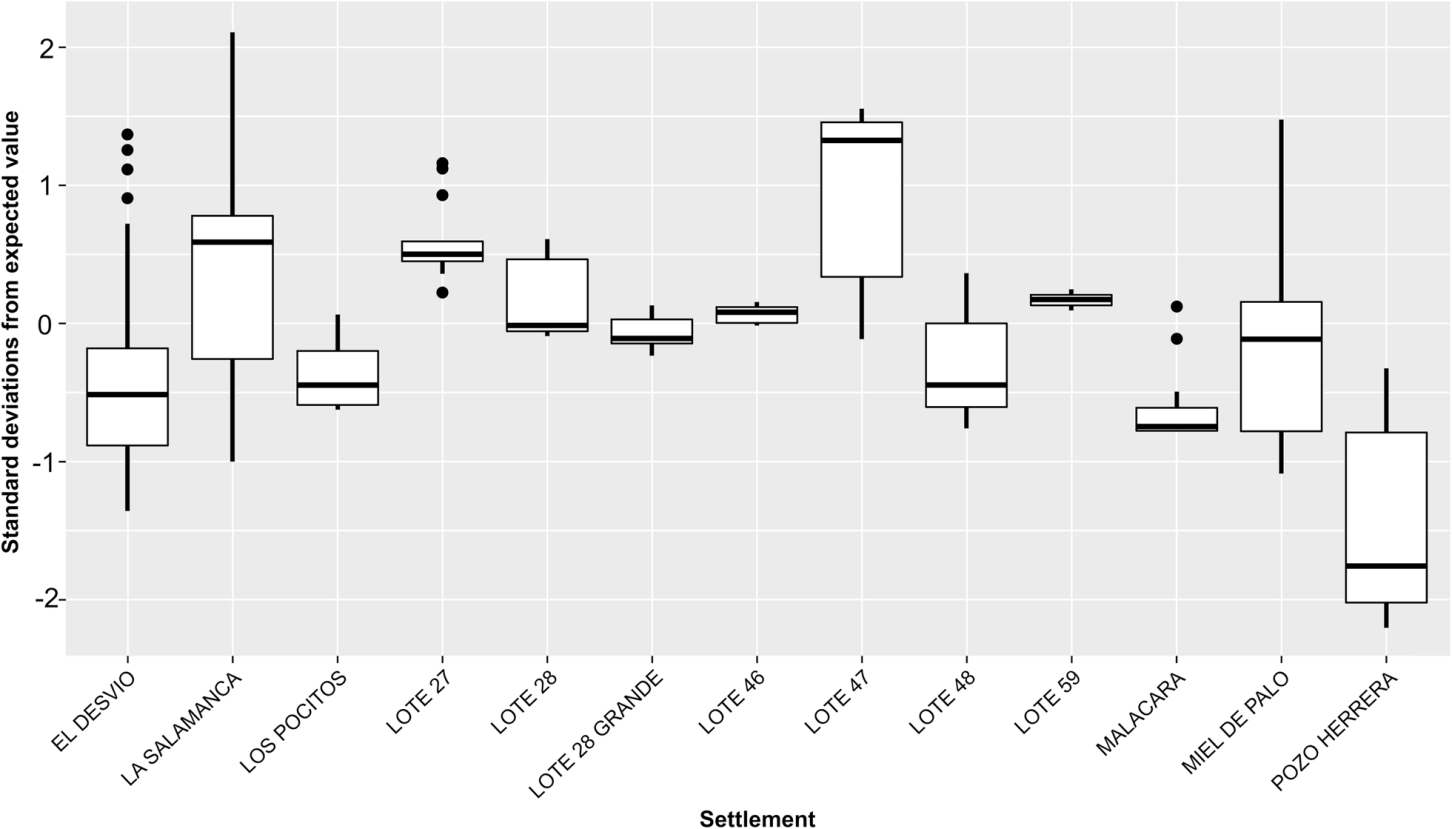
Box plot showing the distribution of the standardized difference (Sdif) of triatomine infestation (2009–2019) in each rural settlement from Añatuya (Santiago del Estero, Argentina). The box represents the interquartile range (IQR), with the central line indicating the median. Black points outside the whiskers represent outliers, defined as values more than 1.5 times the IQR below Q1 or above Q3

Additionally, the infestation rate was not uniform within each settlement, this variability can be observed in Fig. 3. This figure provides a concise view of the distribution of values, allowing visualization of the mean infestation per settlement, intra-settlement variability, and comparison between localities. The *y*-axis in the plot represents the deviation from a homogeneous distribution in standard deviations, where positive values indicate hotspots and negative values indicate cold spots, thus confirming those settlements with high- and low-density infestation, and visualizing the infestation variability within each settlement, such as Miel de Palo, La Salamanca, Pozo Herrera, and Lote 47. Additionally, it helps identify the settlements with the highest mean infestation levels (e.g., Lote 47, La Salamanca, Lote 27).

**Fig. 4:**
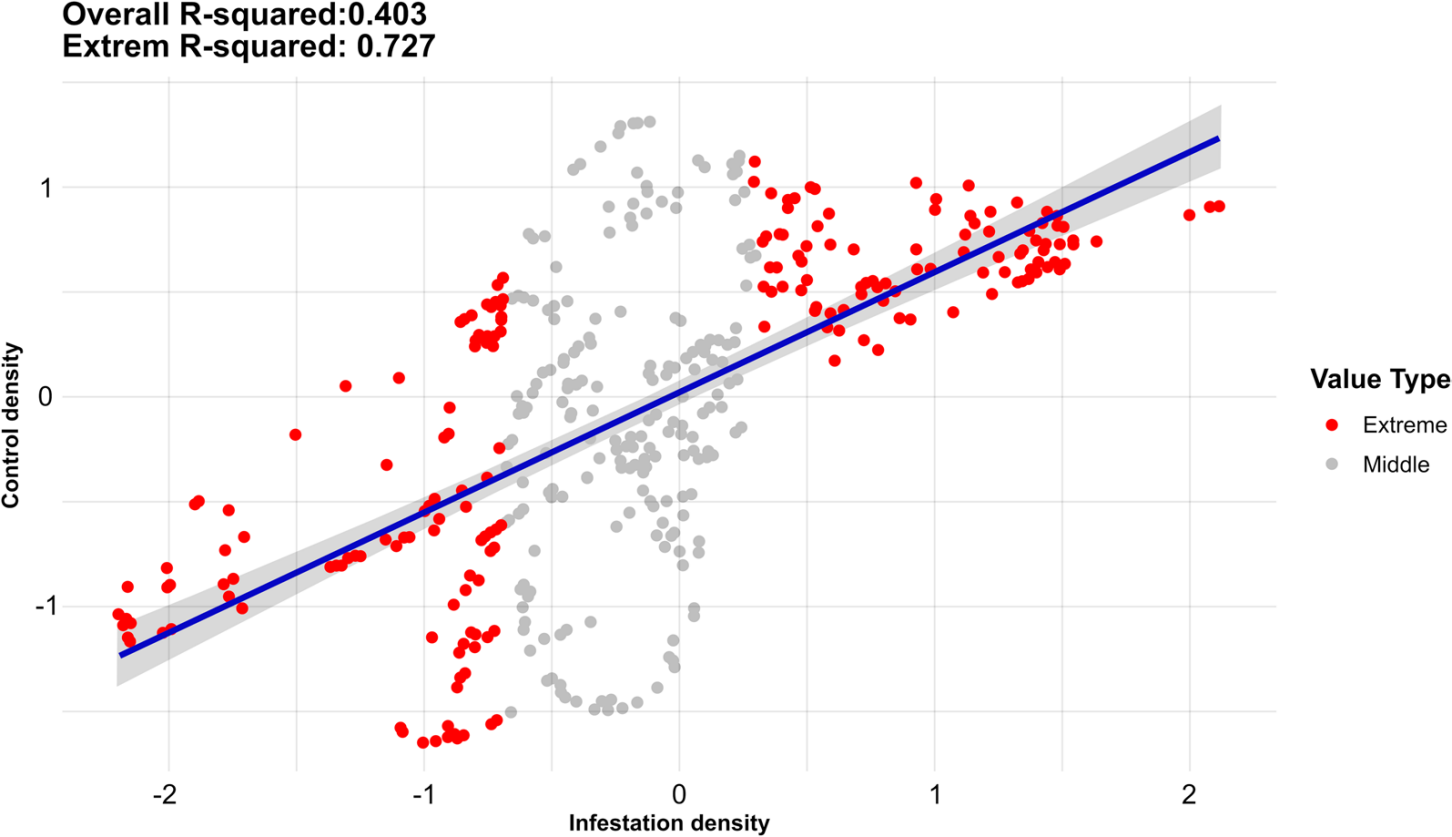
Scatter plot comparing the triatomine infestation density versus the control density (2009–2019) in each rural settlement from Añatuya (Santiago del Estero, Argentina) included in the study. The *R*-squared value has been calculated in two different ways: considering all values (Overall *R*-squared) and only the extreme values (20th–80th percentiles) (Extreme *R*-squared)

Given the standard control measures applied in this area, a scatter plot (Fig. 4) with infestation density (*x*-axis) was plotted against insecticide application density (*y*-axis), to explore the relationship between these two variables per household. Figure 4 shows that insecticide application is neither homogeneous nor follows a regular spatial pattern; insecticide control does not exactly coincide with infestation persistence. An overall Spearman correlation of 0.403 was compared with an *R*-squared value of 0.727 for the extreme values of infestation (red dots, Fig. 4), suggesting that 72.7% of the variability in control density can be explained by infestation density among the extreme values. This higher value indicates a stronger and more consistent linear relationship within the extremes, where both low and high infestation densities align closely with control densities. In contrast, middle-range values exhibit greater variability, likely contributing to the overall reduction in model fit.

Notably, the analysis reveals spatial clusters of households that deviate from the general trend. While our current set of variables does not allow us to clearly identify whether these deviations correspond to under- or over-treatment, their spatial structuring suggests the presence of operational or ecological factors not captured by the present model. From a programmatic standpoint, these outliers could represent priority areas for further investigation, potentially indicating inefficiencies in resource allocation or localized barriers to treatment effectiveness.

### Landscape transformation

**Fig. 5:**
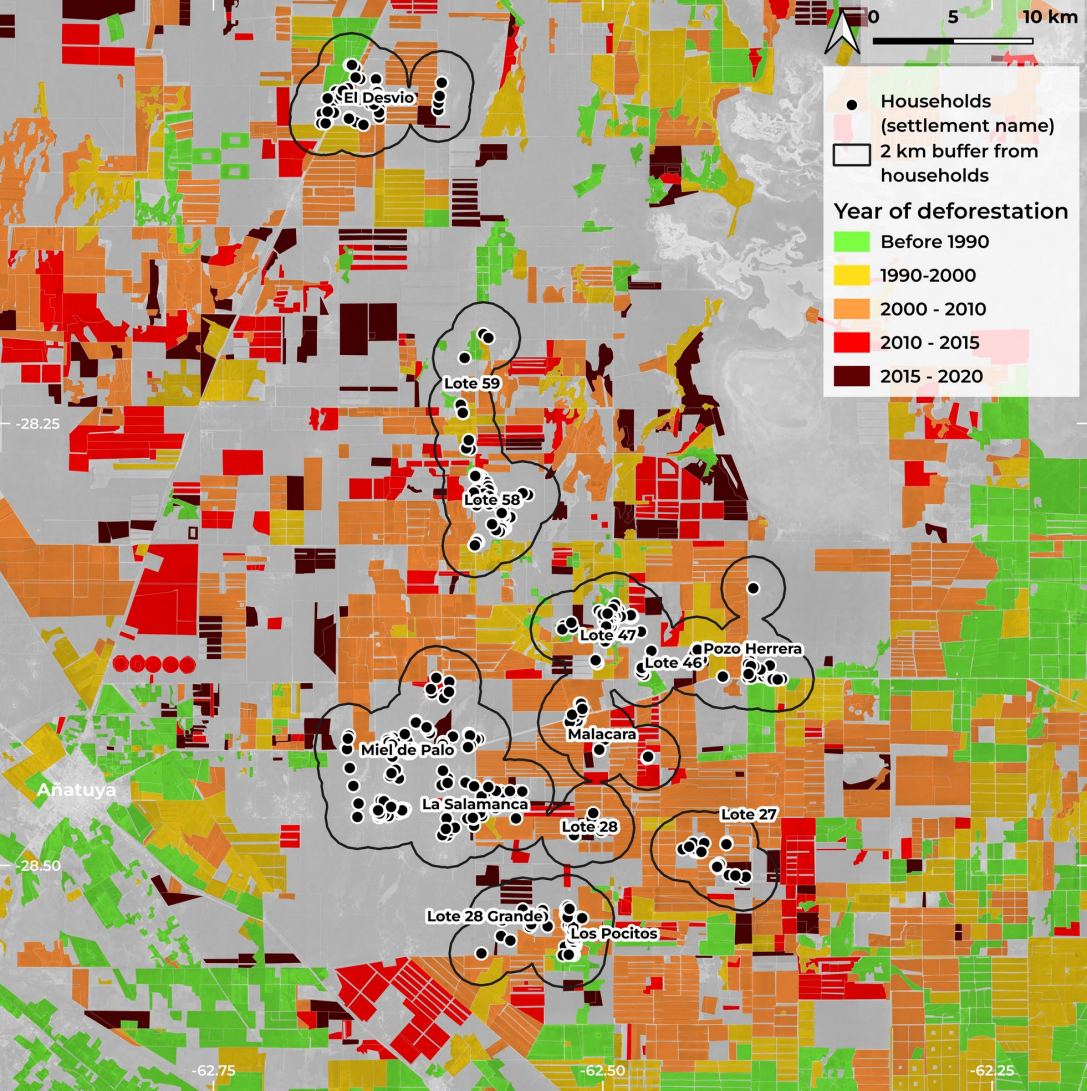
Map of deforestation in Añatuya and surrounding rural areas of Santiago del Estero (Argentina) from 1990 to 2020, within the study area and period of study. The spatial reference unit is a 2 km buffer zone around each household, outlined in black. Black dots indicate household locations. Background satellite imagery from Google Maps © Google Maps, CNES/Airbus, TerraMetrics, 2024)

Figure 5 shows the impact of landscape transformation in the study area, providing a temporal view of deforestation according to the year it occurred. These deforested areas form the basis for calculating statistics related to landscape variables in the analysis. The spatial reference unit for these analyses is the 2 km buffer zones around each household, outlined on the map with a black line. There are areas near the localities where deforestation has been occurring since 1990, but mostly since 2000, with continuous deforestation between 2015 and 2020.

**Fig. 6:**
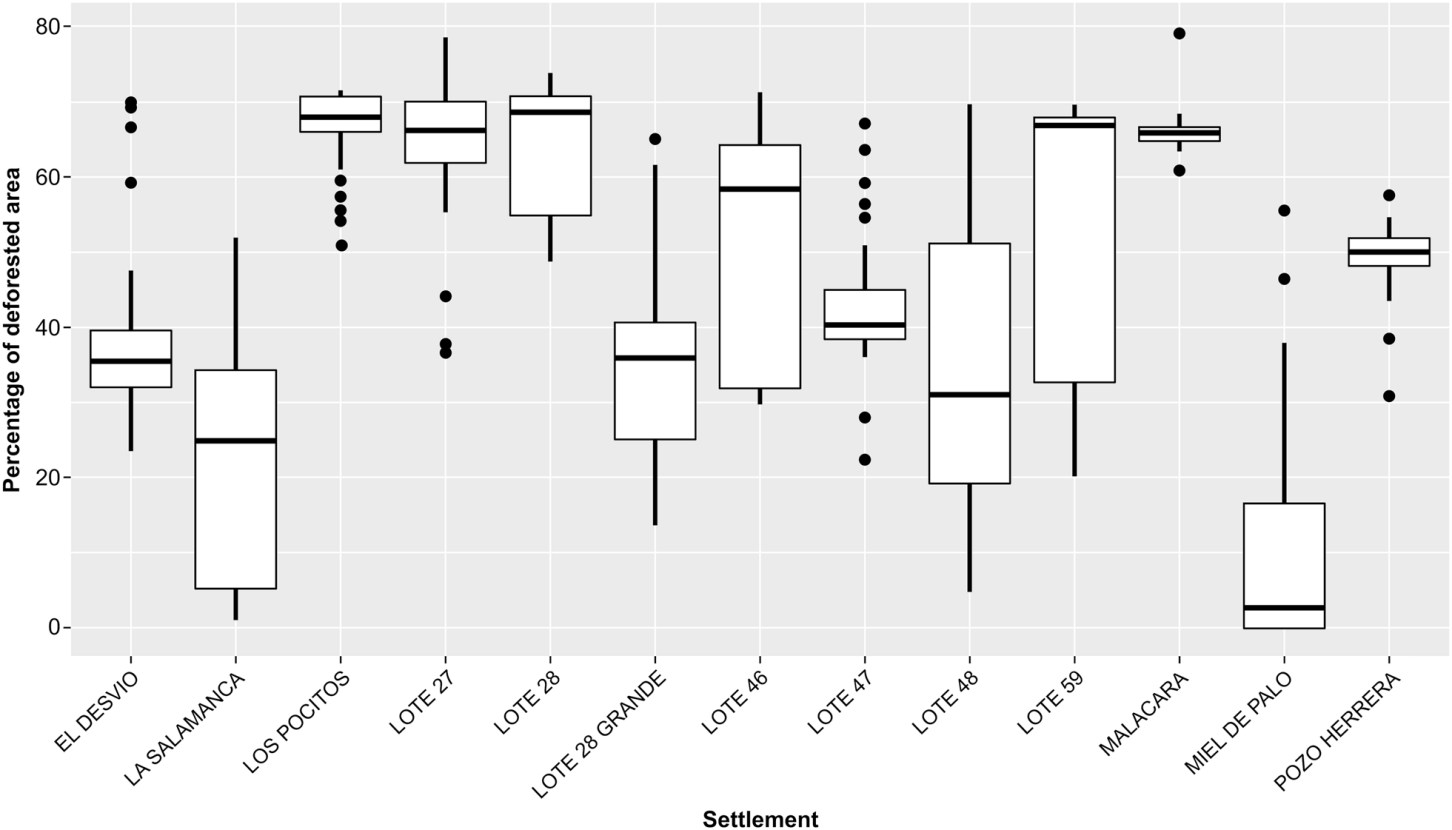
Box plot showing the percentage of deforested area (1990–2020) per rural settlement in the study area (Añatuya, Santiago del Estero, Argentina) within a 2 km buffer around each household

**Fig. 7:**
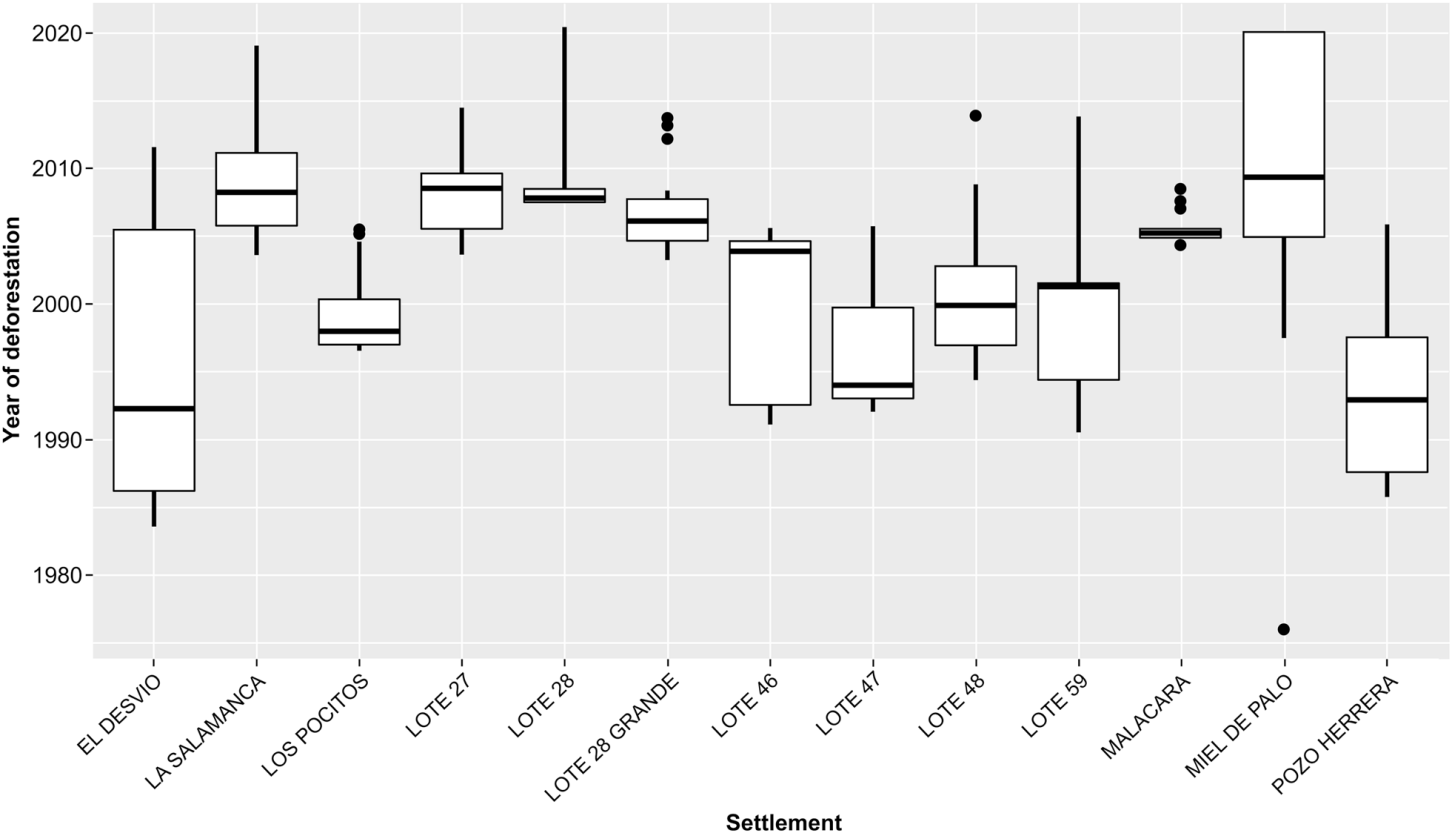
Box plot showing the years of deforestation (1990–2020) in surrounding areas per settlement in the study area (Añatuya, Santiago del Estero, Argentina), within a 2 km buffer around each household

To assess the impact of agricultural transformation within each settlement, a box plot was used (Fig. 6), grouping the data on the percentage of area transformed into agricultural land for each buffer zone (2 km around each household). This analysis allows us to discern the degree to which each settlement has been affected by the expansion of the agricultural frontier, as well as a visualization of the environmental variability within each settlement. The percentage of deforested areas varied significantly, ranging from an average of less than 10% for Miel de Palo to an average greater than 60% in several settlements such as Lote 27, Lote 28, Los Pocitos, Lote 59, and Malacara. Additionally, Fig. 7 shows the average year in which the largest land transformation changes occurred, with El Desvio, Los Pocitos, Lote 47, and Pozo Herrera representing those settlements with large land changes before the year 2000 and the rest occurring during the twenty-first century. Some settlements have suffered deforestation for 10 years or more, including Miel de Palo, La Salamanca, El Desvio, Pozo Herrera, and Lote 46.

### Random forest analysis

To understand and explore factors that might be associated with the observed triatomine infestation in the study area, taking the household as a unit, independent of its grouping within a particular settlement, two random forest models were used. Model 1 explained 94.7% of the observed variability, using six predictor variables, including: household density within the settlement, perimeter of deforested area, years of deforestation, percentage of deforested area, average distance to deforested area, and distance to main roads (Additional File 1: Text S1). To maintain a significance level of 0.1 in the infestation values, a significance filter was applied, thus reducing the sample size from 535 to 377 households. To determine the variables that most influence, and thus explain, the triatomine infestation observed, the analysis of variable importance revealed household density within each settlement and the distance to main roads as explanatory variables (Additional File 2: Fig. S1).

**Fig. 8:**
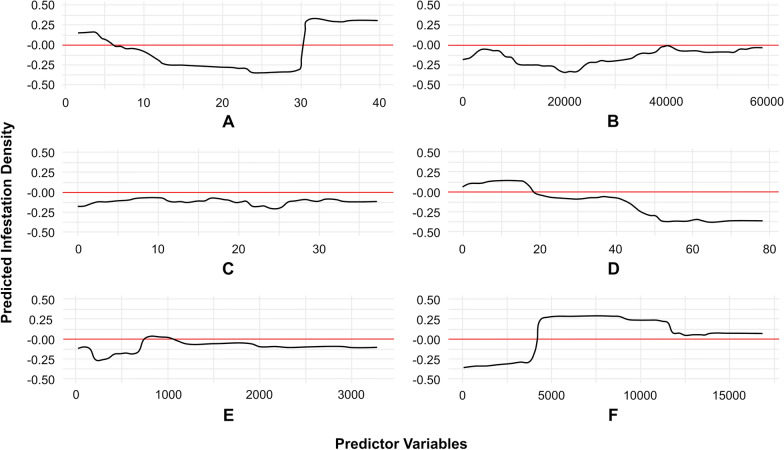
Partial dependence maps from random Forest model 1, illustrating the behavior of predictor variables in the rural settlements from Añatuya (Santiago del Estero, Argentina): **A** household density within the settlement (households/hectare), **B** perimeter of deforested area (m), **C** years of deforestation, **D** percentage of deforested area, **E** average distance to deforested areas (m), and **F** distance to main roads (m)

The partial dependence plots (PDPs) in Fig. 8 show the relationship between the models’ predictor variables, which were found to have nonlinear relation with the spatial pattern of triatomine infestation. Regarding the most important predictor variable in this model, housing density within the settlement (Fig. 8A) showed significant partial dependence with triatomine infestation in areas with both lower and higher household density, while areas with intermediate house density (12–30 per hectare) had lower infestation. However, infestation increased as the distance to main roads increased (Fig. 8F), with lower infestation in households located 0–4000 m from main roads. Additionally, infestation decreased as deforestation increased, showing a direct association (Fig. 8D). Meanwhile infestation increased as the distance to deforested areas increased, up to approximately 1000 m, and then remained stable (Fig. 8E).

**Fig. 9:**
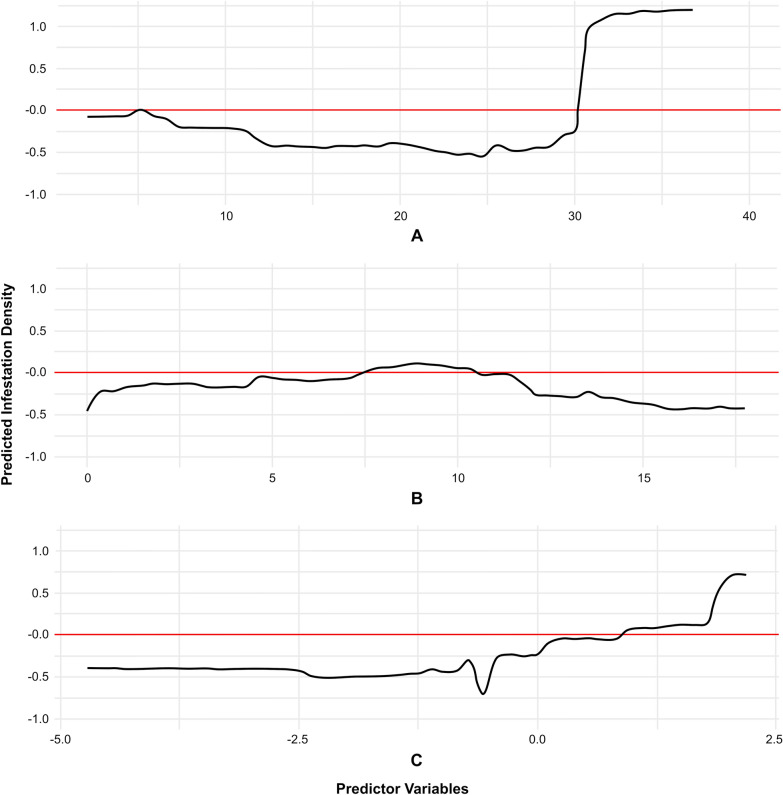
Partial dependence maps from random Forest model 2, illustrating the behavior of predictor variables in the rural settlements from Añatuya (Santiago del Estero, Argentina): **A** household density within the settlement, **B** weighted deforestation, and **C** domestic animal density

Based on the results of the first model, model 2 was designed to be more interpretable, using three predictor variables: household density within the settlement, weighted deforestation (combining percentage of deforested area, years of deforestation, and the average distance to deforested areas), and density of domestic animals (Additional File 3: Text S2). For this model, the sample size was 219 households because not all settlements had registered animal data. Although this model had a lower percentage of explained variance, concretely 83.05%, it is still considerably high. As in model 1, household density within the settlement was the most important variable, followed by weighted deforestation, and animal density (Additional File 4: Fig S2; Additional File 5: Figure S2). In this model, the PDPs show more regular patterns (Fig. 9). For both housing density and animal density, higher values of the variables correspond to higher infestation values. However, the magnitude of deforestation shows greater importance at intermediate values, with a slightly skewed distribution toward lower values (Fig. 9B). Meanwhile, there is an inverse relationship between animal density and deforestation magnitude, which could be explained by space limitations in areas with high agricultural activity, or a shift in local economic activity (i.e., land use). In summary, both random forest models show nonlinear relationships with explanatory variables and highlight the importance of deforestation.

## Discussion

Zoonotic diseases, such as Chagas, involve the spillover of pathogens between species, with transmission driven by factors such as the prevalence of infection in reservoir animals, the rate of contact between humans and vectors, and the probability that contact will cause infection [49]. Human activities, particularly land use changes, significantly alter these factors, influencing the abundance and distribution of reservoir hosts, vectors, and ultimately the likelihood of human exposure [50].

The Gran Chaco Region of Argentina has suffered many landscape changes due to deforestation for agricultural and cattle ranching purposes [3, 51, 52]. These changes have had a profound impact on local flora and fauna [53], and specifically on the sylvatic cycle of *T. cruzi*. Studies in Santiago del Estero [16] and other areas [30, 54, 55] indicate a reduction in the prevalence and incidence of *T. cruzi* in sylvatic hosts, decreasing the risk of spillover to domestic environments. Furthermore, a recent study in the same study area detailed herein shows a decrease in animal diversity, including the lack of marsupials, key reservoirs of *T. cruzi* [54], as well as undetectable levels of infection in sampled mammals such as skunks and armadillos [29].

However, deforestation does not always uniformly reduce the risk of transmission. In some areas, environmental degradation is associated with an increase in domestic triatomine populations, particularly *T. infestans*, a species adapted to human dwellings. While *T. infestans* is primarily domiciliary, landscape changes can still influence the establishment and colonization of *T. infestans* [16, 32]. In less anthropic landscapes, the dynamics of secondary vector species, such as *Triatoma garciabesi* have shown higher density [56]. This has also been observed in patches of native trees within a Monte ecoregion, with the presence of secondary species and *T. infestans* adults close to the households [57, 58]. This indicates that landscape structure plays a key role in infestation dynamics in this area with allochthonous *T. infestans*.

The current study focused on analyzing these factors at a local spatial scale, which enabled an analysis of the infestation dynamics present in the households grouped into 13 different rural settlements of Añatuya that have been under continuous surveillance and control since 2005. Because of this difference in scale, the observed phenomena such as deforestation or the distribution of households and the persistence of *T. infestans* could present different patterns than those observed at a larger scale.

### Influence of house configuration on infestation patterns

The configuration and disposition of households within the modified landscape matrix also influences infestation risk, and it does not depend on the location of the household within a given settlement. When analyzing the expected and observed infestation distribution (Fig. 2), hotspots and cold spots of a smaller scale than a specific settlement can be identified, such as in Miel de Palo or La Salamanca. Spatial studies reveal that house density, proximity to agricultural plots, and the presence of peridomestic structures are significant factors in explaining patterns of triatomine infestation [12, 20, 59]. Households located near forested areas, as opposed to those surrounded by cultivated land, tend to experience higher infestation rates [60]. This is consistent with findings from other regions, where areas of persistent infestation were associated with greater forest cover and lower agricultural activity [16]. The variability in landscape matrices—ranging from dense forest to open farmland—affects the microenvironment, which in turn influences the colonization and persistence of allochthonous triatomine populations.

The results from surveillance efforts in rural Argentina also emphasize the role of microenvironmental factors. Houses in areas with a high density of households tend to exhibit regular infestation patterns over time, either high or low, suggesting that the configuration of the local environment plays a crucial role in the colonization and persistence of *T. infestans* [22, 60]. Persistent infestation sites could serve as important guides for control programs, helping determine when to shift from active to passive surveillance [22].

### The role of surveillance and control programs

The long-term impact of sustained vector surveillance and control programs has been critical in reducing *T. cruzi* transmission in the Gran Chaco region. Studies have shown that ongoing surveillance combined with vector control strategies, including insecticide spraying and housing improvements, can lead to sustained reductions in infestation rates [18, 21, 23]. In areas such as Añatuya, Santiago del Estero, the introduction of house modifications, such as wire-fenced compounds and the relocation of houses, has further contributed to reducing vector populations [35]. The presence of animals, particularly chickens which are refractory to *T. cruzi* infection, also plays a role in reducing human–vector contact and thus transmission risk [21].

However, the efficiency of control efforts can vary on the basis of local environmental conditions and the persistence of sylvatic triatomine populations [17, 18, 27, 61]. For example, sylvatic foci of *T. infestans* have been identified in certain areas, posing a reinfestation risk, particularly in regions undergoing land-use changes [62]. This highlights the need for tailored vector control strategies that account for both domestic and sylvatic infestation dynamics.

### Broader regional considerations and spatial scale

While this study focuses on the local scale of Añatuya, broader regional patterns should also be considered. In areas of the Gran Chaco that have undergone more extensive deforestation, a more direct relationship between reduced wildlife diversity and disruption of the *T. cruzi* sylvatic cycle is likely to occur [4]. In such regions, housing configuration within the altered landscape could reveal more consistent patterns of triatomine infestation and recolonization, offering a wider perspective on vector dynamics and control strategies.

The reduction in sylvatic mammals, particularly marsupials, which serve as important reservoirs for *T. cruzi*, has been observed in various regions, supporting the idea that environmental degradation limits the spillover of the parasite into domestic settings [30, 54]. Here the inverse relation between deforestation and farm and domestic animal density reflects land use change for agricultural uses, supporting the limitation of the spillover hypothesis, underscoring the importance of integrated control programs that consider both vector dynamics and broader ecological changes, such as deforestation and habitat fragmentation.

## Conclusions

In conclusion, the findings of this study suggest that both local and regional landscape transformations have a significant impact on the presence of *T. infestans* and dynamics of *T. cruzi* transmission. The configuration of houses, combined with environmental changes such as deforestation, play a pivotal role in determining the spatial patterns of triatomine infestation. Sustained surveillance and control efforts conducted in this rural area since 2005 [35], have shown an overall reduction in household triatomine infestation, both in the intra- and peridomicile. Additionally, the absence of a sylvatic cycle of *T. cruzi* and a lack of *T. cruzi* infection in captured triatomines and sampled domestic and sylvatic animals from the same area has been reported [29].

Throughout the Gran Chaco region, deforestation for agricultural purposes has been the main environmental alteration [63, 64]. Considering that spatial clustering of house infestation is related to this alteration, together with other local variables (such as the presence of animals and the configuration of rural dwellings), targeting control strategies in areas with recent or more deforested areas, with higher density of houses and domestic animals per km^2^ would be a recommendable strategy. Additionally, the data analyzed herein, with respect to the alteration of the sylvatic and domestic cycles of *T. cruzi*, need to be considered to guide future strategies for continuous monitoring of the situation to advance on diagnosis and treatment of infected individuals and to collect the data required by guidelines from the Pan American Health Organization (PAHO) [65] to certify this area as free of vector transmission.

## Supplementary Information

Additional file 1.

## Data Availability

No datasets were generated or analyzed during the current study.

## Abbreviations

CD: Chagas disease
NTDs: Neglected tropical diseases
*T. cruzi*: *Trypanosoma cruzi*
*T. infestans*: Triatoma infestans
KDE: Kernel density estimation
S&C: Entomological surveillance and control
SDif: Standardized difference
LISA: Local indicator of spatial association
RFMs: Random forest models
IncNodePurity: Increase in node purity
PDPs: Partial dependence plots
IQR: Interquartile range
NDVI: Normalized difference vegetation index
PAHO: Pan American Health Organization
