# Supplementary material for: Association between landscape transformation and the Chagas disease vector dynamics in a rural area with continuous surveillance and control
Source: Parasit Vectors. 2025 Jun 2;18:203. doi: 10.1186/s13071-025-06849-1 (PMC12131463; doi:10.1186/s13071-025-06849-1)
Supplement: Supplementary file 1 — Additional file 1. [file 13071_2025_6849_MOESM1_ESM.docx]

**Supplementary Material**

**Additional File 1: Text S1**

Output of the RStudio software for Model 1:

Call: randomForest (formula = Inf_density ~ A + B + C + D + E + F, data = DB, mtry = 2)

Type of random forest: regression

Number of trees: 500

No. of variables tried at each split: 2

Mean of squared residuals: 0.04681075

% Var explained: 94.73

Variable Definitions: (Inf_density) Infestation density; (A) Household density within the settlement (households/km²); (B) Perimeter of deforested area (meters); (C) Years of deforestation; (D) Percentage of deforested area; (E) Average distance to deforested areas (meters); (F) Distance to main roads (meters); (DB) Database

**Additional File 2: Fig. S1**

**
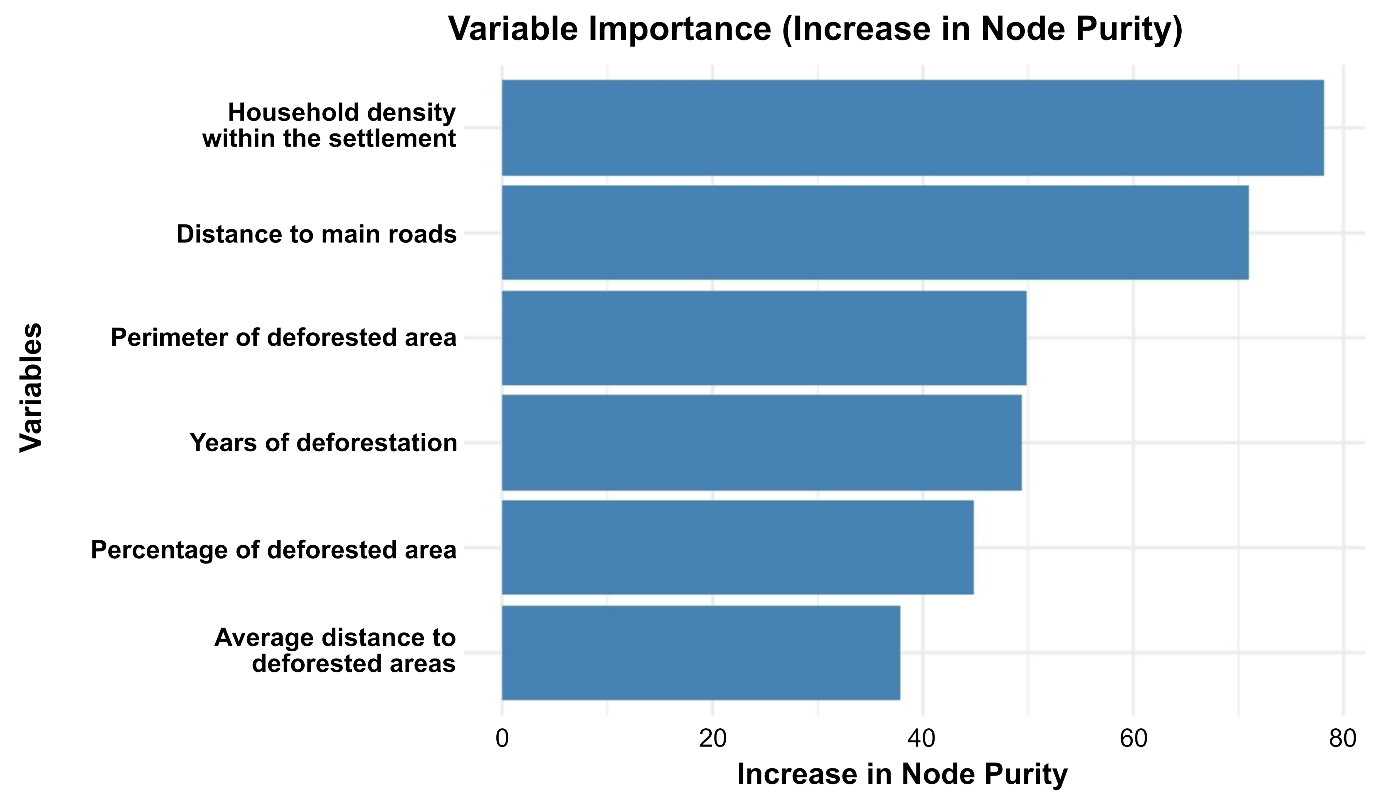
**

**Figure S1**. Bar graph ranking the variables used in model 1 of the random forest analysis based on the value of Node Purity.

**Additional File 3: Text S2**

Output of the RStudio software for Model 2:

Call: randomForest (formula = Inf_density ~ A + B + C, data = DB, mtry = 2)

Type of random forest: regression

Number of trees: 500

No. of variables tried at each split: 2

Mean of squared residuals: 0.1358922

% Var explained: 83.05

Variable Definitions: (Inf_density) Infestation density; (A) Household density within the settlement; (B) Weighted deforestation; (C) Domestic animal density; (DB) Database

**Additional File 4: Fig. S2**


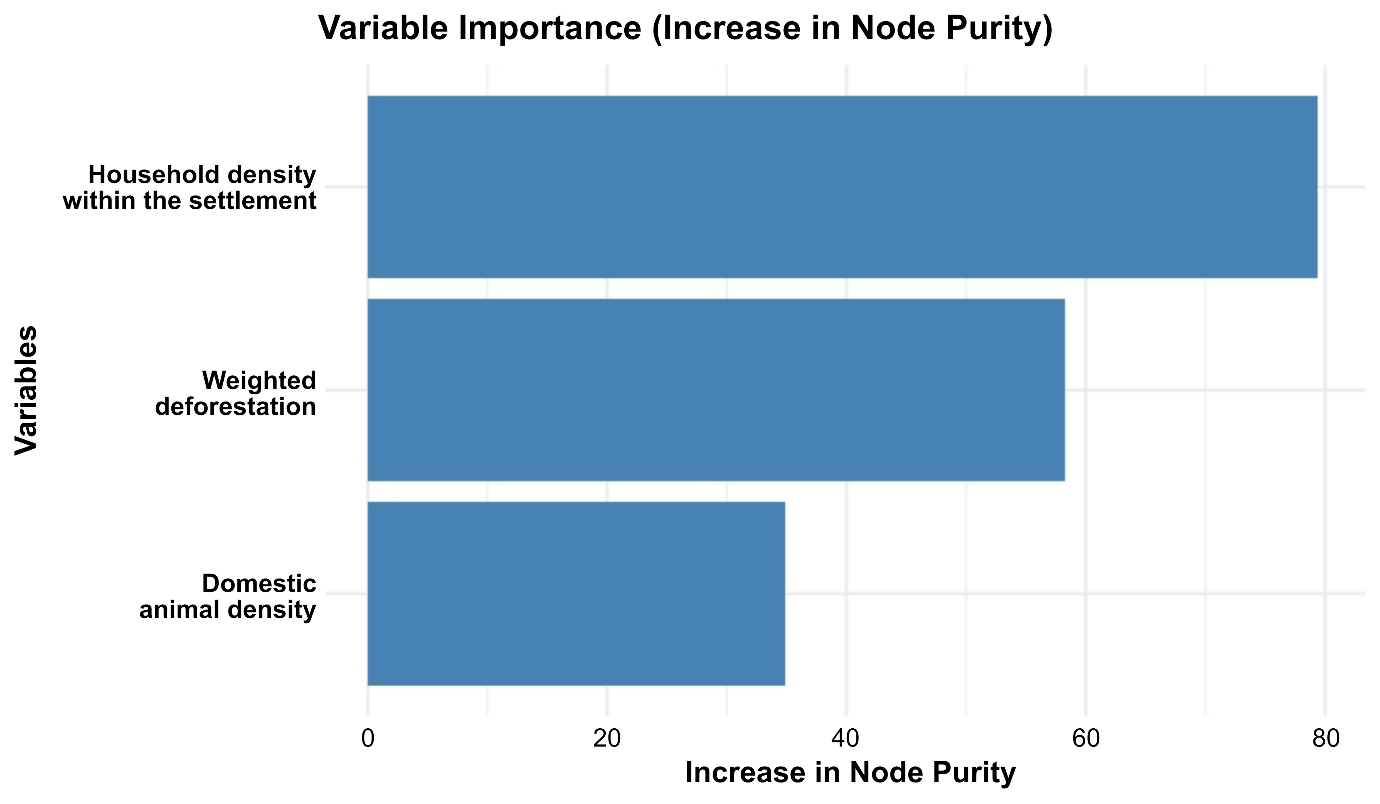


**Figure S2**. Bar graph ranking the variables used in model 2 of the random forest analysis based on the value of Node Purity.

**Additional File 5: Figure S3**


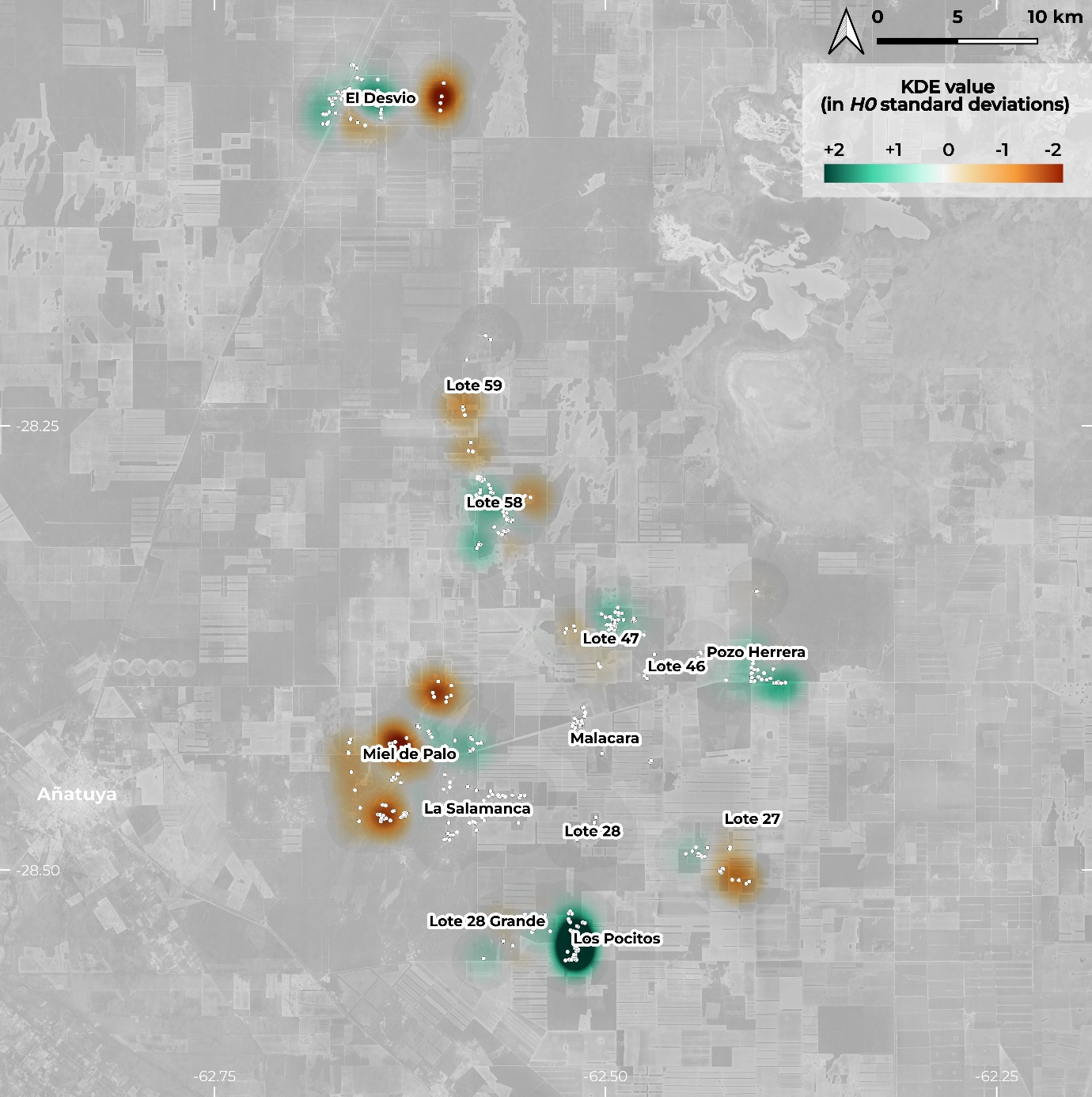


**Figure S3**. Map of weighted animal density estimated using kernel density estimation (KDE). Values represent deviations from the expected density (H0), with positive values indicating higher than expected density and negative values indicating lower than expected density. Created using QGIS with background imagery from Google Maps via the QuickMapServices plugin (version 0.19.36). © 2024 Google. Map data 2024 Google.
